# Nose to brain delivery of mirtazapine via lipid nanocapsules: Preparation, statistical optimization, radiolabeling, in vivo biodistribution and pharmacokinetic study

**DOI:** 10.1007/s13346-024-01528-7

**Published:** 2024-02-20

**Authors:** Mennatullah M. Ibrahim, Emad B. Basalious, Mohamed A. El-Nabarawi, Amal IA. Makhlouf, Marwa Eid Sayyed, Ismail Taha Ibrahim

**Affiliations:** 1 Department of Pharmaceutics and Industrial Pharmacy, Faculty of Pharmacy, Cairo University, Cairo, Egypt; 2 Department of Pharmaceutics and Industrial Pharmacy, Faculty of Pharmacy, October University for Modern Sciences and Arts (MSA), Cairo, Egypt; 3 Radio Labeled Compounds Department, Hot Labs Centre, Egyptian Atomic Energy Authority, P.O. Box 13759, Cairo, Egypt; 4 Faculty of Pharmacy, Albayan University, Baghdad, Iraq

**Keywords:** Mirtazapine, Intranasal, Lipid nanocapsules, Brain, ^131^I

## Abstract

Mirtazapine (MZPc) is an antidepressant drug which is approved by the FDA. It has low bioavailability, which is only 50%, in spite of its rapid absorption when orally administered owing to high first-pass metabolism. This study was oriented towards delivering intranasal (IN) mirtazapine by a direct route to the brain by means of preparing lipid nanocapsules (LNCs) as a targeted drug delivery system. MZP-LNCs were constructed by solvent-free phase inversion temperature technique applying D-Optimal mixture design to study the impact of 3 formulation variables on the characterization of the formulated nanocapsules. Independent variables were percentage of Labrafac oil, percentage of Solutol and percentage of water. Dependent variables were particle size, polydispersity index (PDI), Zeta potential and solubilization capacity. Nanocapsules of the optimized formula loaded with MZP were of spherical shape as confirmed by transmission electron microscopy with particle diameter of 20.59 nm, zeta potential of − 5.71, PDI of 0.223 and solubilization capacity of 7.21 mg/g. The in vivo pharmacokinetic behavior of intranasal MZP-LNCs in brain and blood was correlated to MZP solution after intravenous (IV) and intranasal administration in mice. In vivo biodistribution of the drug in mice was assessed by a radiolabeling technique using radioiodinated mirtazapine (^131^I-MZP). Results showed that intranasal MZP-LNCs were able to deliver higher amount of MZP to the brain with less drug levels in blood when compared to the MZP solution after IV and IN administration. Moreover, the percentage of drug targeting efficiency (%DTE) of the optimized MZP-LNCs was 332.2 which indicated more effective brain targeting by the intranasal route. It also had a direct transport percentage (%DTP) of 90.68 that revealed a paramount contribution of the nose to brain pathway in the drug delivery to the brain.

**Graphical Abstract:**

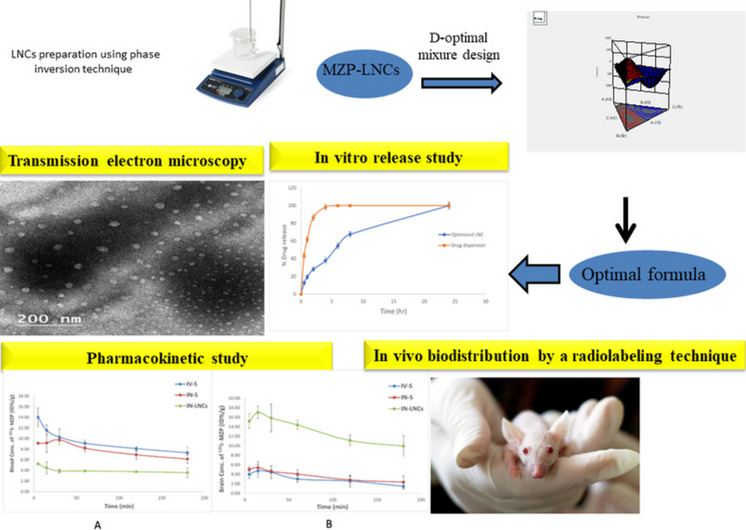

## Introduction

Anxiety and depression are the most frequent mental illnesses in many countries [1]. Generalized anxiety disorder (GAD) is described by extreme, too much anxiety and fear over ordinary life happenings with no clear reasons, whereas depression is characterized by a condition of low mood and dislike of activity which may disturb a person’s thinking, actions, emotions and physical state. Depressed people appear to have a bad mentality, with strong feelings of sorrow, anxiousness, suicidal thoughts, disrupted sleeping, and maybe a weakened passion for joyful activities during most of their days. It may also lead to many physical and mental problems, in addition to poor productivity at work and at home [2, 3]. There are nearly 300 million patients living with depression all over the world which indicates its global prevalence [1, 4]. At its worst, depression may lead to suicide. Medical care professionals might offer psychotherapy as well as additional drugs such as tetracyclic antidepressants, tricyclic antidepressants (TCAs) and selective serotonin reuptake inhibitors (SSRIs) based on the sequence and severity of the episodes of depression throughout time [5].

Treatment of CNS (central nervous system) diseases is difficult due to many difficult barriers for efficient drug delivery. Despite potency of CNS acting drugs, their clinical failure is common, and this is due to limitations in the drug delivery strategy rather than the lack of pharmacological effectiveness of these drugs. Despite its potency, the drug is rendered ineffective due to its disability to pass the blood–brain barrier (BBB) and enter the brain to perform its pharmacologic effect [1, 6, 7]. Therefore, researchers are investigating innovative ways for enhancement drugs delivery to the CNS and targeting them to the brain.

Mirtazapine (MZP) is an antidepressant used to manage mild to severe depression, which is frequently accompanied with symptoms of anxiety. It is a tetracyclic antidepressant that acts on certain serotonergic and noradrenergic receptors. Furthermore, it is used for the treatment of anxiety through rising central noradrenergic and serotonergic (5 HT1) neurotransmission. It works largely as a strong antagonist at postsynaptic 5 HT2 and 5 HT3 (serotonergic) receptors as well as central noradrenergic receptors. It is the only tetracyclic antidepressant authorized by the FDA for treating depression [3, 5]. MZP has a low solubility in water (1.1 mg/mL) [8] and its partition coefficient is 2.9 [3, 9]. MZP is usually taken orally, but this is linked with low bioavailability of about 50% and limited clinical efficacy as it undergoes hepatic first pass metabolism [1, 10]. Additionally, it can cause many systemic adverse effects including tiredness, sleepiness, and dizziness [11].

In the light of above facts, MZP should be delivered via different routes to avoid first pass metabolism and improve its bioavailability. Of these routes, drug delivery from the nose to brain has a prominent interest. Intranasal (IN) drug administration is a direct and non-invasive route to deliver drugs to the brain via trigeminal nerve and olfactory nerve passageways evading the blood brain barrier (BBB) [12]. It also can bypass hepatic metabolism and decrease several off-target adverse effects, and therefore, it appears as an interesting administration route [13, 14]. Nasal cavity is supplied by six branches of arteries that may improve drugs systemic absorption; furthermore, existence of the olfactory area may offer a way to target the brain directly [15]. The combination of all those factors along with high surface area in the nasal cavity may help to improve medication absorption. Numerous benefits of drug encapsulation involve prolonged release, solubilization of drugs that are poorly soluble, avoiding precipitation of the drug upon diluting it and preventing destabilization. Drugs can be encapsulated in liposomes, microspheres and nano-carriers [16–18]. Many nano-carriers are absorbed directly to the brain along the olfactory nerve pathway following IN delivery. Successful IN delivery of several drugs including nimodipine [19], clonazepam [20], diazepam [21], tacrine [22], zolmitriptan [23], sumatriptan [24] and cabergoline [25] as nano-carrier systems have been achieved leading to enhanced drug absorption. Many nanocarriers have been applied for the intranasal delivery of antidepressant drugs in an attempt to protect them from protein degradation, enhance their olfactory mucosal uptake and CNS utilization and prolong their half-life. These nanocarriers were either polymer-based like PLGA nanoparticles [26], chitosan nanoparticles [27] and alginate nanoparticles [28], or lipid-based like liposomes [29, 30], transferosomes [31] and elastosomes [32].

Lipid nanocapsules (LNCs) are nanocarriers consisting of a tensioactive shield around a lipophilic core [33]. They are prepared by solvent-free method using the principle of phase inversion during the heat treatment of water and oil. LNCs can better encapsulate lipophilic drugs. Furthermore, the method of preparation of LNCs is simple and repeatable and doesn’t need the use of large amount of surfactant and cosurfactant or organic solvent; therefore, LNCs are considered to be safe [34]. Also, the study of Thomas et al. 2013 [35] showed that lipid nanocapsules could be scaled up. Moreover, lipid nanocapsules are stable and have particle size in the nano range. All these features make LNCs a better choice comparing to nano and microemulsions, liposomes and other nanocarriers [36].

There are numerous pharmaceutical applications that have studied lipid nanocapsules utilizing various administration routes such as parenteral [37], dermal [38] and oral [39, 40]. Nevertheless, LNCs have not yet been investigated for IN administration to deliver drugs to the brain directly. There are two pathways for nano-carriers to deliver the drug into brain following IN administration. First is the olfactory pathway in which the nano-carriers transfer the drug directly to the brain across the olfactory nerve bypassing BBB (nose-brain delivery) [41–43]. Second is the systemic pathway in which the drug reaches the systemic circulation then passes through the BBB into the brain bypassing nasal epithelium (nose-blood–brain delivery) [44]. Contribution of the two pathways in delivery of the drug to the brain following IN administration of lipid nanocapsules is still unclear.

To assess in vivo biodistribution, generally, radiolabeling of drugs [45] or the best formulation [46] using a suitable radiolabeling indicator [47] is often utilized. Therefore, the point of this work is to formulate MZP-loaded LNCs (MZP-LNCs) utilizing the solvent-free phase inversion temperature method. The target is to deliver MZP directly to the brain through the nose to decrease its systemic entrance and realize direct nose to brain transport. Thus, enhancement of MZP brain bioavailability is obtained. MZP-loaded LNCs were evaluated for their particle diameter, polydispersity index (PDI), zeta potential and solubilization capacity. The optimum formulation was then assessed for its in vitro drug release and images were obtained by transmission electron microscopy to explore its morphological features. In vivo biodistribution of the optimum formula was evaluated in mice using mirtazapine labeled with radioactive iodine (^131^I-MZP). The pharmacokinetic behavior of IN radioiodinated MZP-loaded LNC in the brain and blood was contrasted with radioiodinated MZP solution following intravenous and intranasal administration in mice.

## Materials and methods

### Materials

Mirtazapine (MZP) was a kind gift by Mash Premiere for Pharmaceutical Industry, Cairo, Egypt. Sodium chloride and cellulose dialysis membrane tubing were procured from Sigma Aldrich, St. Louis, MO, USA. Labrafac™ Lipophile WL1349 was kindly provided by Gattefosse, France. Solutol^®^HS 15 was donated by BASF, Germany. Lipoid^®^S75 was obtained from Lipoid Gmbh, Ludwigshafen, Germany. Potassium dihydrogen phosphate, disodium hydrogen phosphate and ethanol were purchased from El-Nasr Pharmaceutical Co., Cairo, Egypt. Iodine-131 was supplied by the radioisotope production facility of the Egyptian Atomic Energy. N chlorosuccinamide 98% (molecular weight, 116.059 g/mol) was obtained from Chem-Lab industriezone, Cairo, Egypt. All the remaining reagents and chemicals were of analytical grade and used as received.

### D-optimal mixture design for model construction

Utilizing Design Expert 10 software (Stat-Ease, Inc., USA), D-Optimal mixture design has been constructed for assessing the single and combined impacts of the three constituents of the formulation. The percentage of Labrafac oil, Solutol HS (surfactant) and water were the three independent factors (X_1_, X_2_ and X_3_ respectively). The three constituent combinations had a 5 g overall weight. Particle size, zeta potential, polydispersity index and solubilization capacity (Y_1,_ Y_2,_ Y_3_ and Y_4_, respectively) were the four measured responses. For obtaining the optimum statistical parameters, including predicted residual sum of squares (PRESS), the multiple correlation coefficient (*R*^2^) and adjusted multiple correlation coefficient (adjusted *R*^2^), the special quartic, reduced quadratic and reduced cubic models have been selected as the most fitted mathematical models to represent the optimal mixture design. On the basis of initial study, the field of every variable was determined. The upper and lower limits of the three factors as well as the dependent variable constraints are illustrated in Table 1. MZP concentration was held constant during the preparation of MZP -LNCs at 1 mg/mL concentration.

**Table 1:** Factors and Responses of D-optimal mixture design for formulation of MZP-LNCs

Factors	Level	
	Lower (%)	Upper (%)
X_1_: Labrafac (oil)	10	35
X_2_: Solutol (surfactant)	20	50
X_3_: water	40	70

Responses	Desirability constraint	
Y_1_: Particle size	Minimize	
Y_2_: Zeta potential	Maximize	
Y_3_: Polydispersity index	Minimize	
Y_4_: Solubilization capacity	Maximize	

### Formulation of MZP-LNCs

Preparation of MZP-LNCs was done adopting solvent-free phase inversion-temperature method as described in the study of Heurtault et al., 2002 [33]. The described quantity of Labrafac oil and 10 mg of MZP were put in a beaker. To solubilize MZP, the beaker was allowed for heating. The assigned amounts of Solutol^®^ HS, Lipoid S75, sodium chloride and distilled water were placed in the beaker. Sodium chloride was maintained at constant concentration of 1.75%W/W while Lipoid S75 was maintained at constant concentration of 1.5%w/w. Based on the formulae given in Table 2**,** the quantity of the three components, Solutol^®^ HS, water and Labrafac was calculated to complete to 5 g total weight. Then the beaker was held on magnetic stirrer until sodium chloride and Lipoid S75 dissolved. The beaker was heated to 70 °C before being cooled to 40 °C. Three cycles of heating and cooling were applied on the mixture in the beaker. In the last cycle, and upon cooling to 40 °C, 5 mL of cold water at 2 °C were suddenly added to the mixture which was then magnetically stirred for 10 min [33, 36, 48].

**Table 2:** Composition and characterization of the D-optimal mixture design for formulation of MZP-LNCs

Formula number	Labrafac %	Solutol %	Water %	Particle size (nm)	Zeta potential (mV)	PDI	Solubilization capacity (mg/g)
	X_1_	X_2_	X_3_	Y_1_	Y_2_	Y_3_	Y_4_
1	10	50	40	20.59	− 5.71	0.223	7.21
2	19.41	30.13	50.45	29.98	− 5.5	0.05	3.66
3	17.25	20	62.75	36.71	− 3.56	0.072	2.51
4	27.36	25.11	47.53	44.67	− 3.02	0.057	4.1
5	10	27.59	62.41	21.68	− 6.67	0.08	2.72
6	24.97	20	55.03	52.68	− 3.95	0.06	3.11
7	35	23.5	41.5	131.5	− 5.66	0.171	4.71
8	21.73	38.27	40	28.41	− 4.9	0.06	5.88
9	10	42.55	47.45	19.51	− 4	0.145	4.51
10	28.73	31.27	40	40.49	− 4.17	0.069	4.98
11	19.41	30.13	50.45	28.88	− 6.1	0.06	3.71
12	35	23.5	41.5	128.4	− 5.26	0.236	4.69
13	10	20	70	24.33	− 7.6	0.076	2.21
14	19.41	30.13	50.45	30.65	− 5.9	0.059	3.51
15	21.73	38.27	40	29.06	− 5.75	0.102	5.91
16	10	35.14	54.86	20.13	− 3.16	0.1	3.22
17	10	50	40	18.74	− 6.3	0.176	6.89

### Characterization of the prepared MZP-LNCs

#### Particle size, PDI and Zeta potential determination

For the measurement of particle size, zeta potential and PDI at 25 °C, Malvern Zeta Sizer (ZS) (Nano ZS, Malvern Instruments Ltd., Malvern, UK) was used depending on dynamic light scattering technique. The formulated MZP-LNCs were diluted with distilled water (1:100 V/V) to obtain uniform dispersion with good scattering intensity [48, 49]. Three replicate measurements were recorded to ensure reproducibility.

#### Solubilization capacity

Solubilization capacity is the efficacy of plain lipid nanocapsules to dissolve MZP and make it solubilized [48]. Procedures were done as follows: An excess amount of 20 mg MZP was added to 2 g of each formula listed in Table 1 at amber glass containers that were shaken for 24 h at 37 ± 2 °C in a thermostatically controlled shaker. Samples were filtered then; 0.5 g of the filtrate was mixed with 50 ml ethanol in a volumetric flask and sonicated at 50 °C for 6 min. The solubilized quantity of MZP in each formula was measured spectrophotometrically at λ_max_ = 293 nm utilizing a proper calibration curve of MZP in ethanol [10, 50].

### Optimization of MZP-LNCs formulation

Desirability function and numerical optimization were applied to optimize the composition of the formulated MZP-LNCs. The composition of the optimized MZP-LNCs was determined using Design Expert 10 software after establishing the criteria for each response as revealed in Table 1. The goal was to establish LNCs with minimized particle size, PDI and maximized solubilization capacity. The desirability function is a method for determining the best measurements for the independent factors, firstly, by assessing the desirability index for every dependent variable and, finally, to merge all responses in a single desirability function that ranges from 0 to 1 to represent the best values of the independent variables [49, 51].

### Evaluation of the optimized MZP-LNCs

#### Transmission electron microscopy (TEM)

The morphologic characters and particle size of the optimized MZP-LNCs were determined using TEM (JEM-2100, Jeol, Tokyo, Japan). Distilled water was utilized to dilute 100 μL sample to 10 mL. An amount of 50 μL of the diluted sample was permitted to dry on copper grid for thirty minutes. The dried sample was subsequently treated with 50 μL of 2% phosphotungestic acid (PTA stain) at pH 7 and allowed to sit for 30 min in order to permit for the proper staining procedure. Then, the sample was examined by TEM operated at 80 kv at 30,000 times of magnification power [52].

#### In vitro release study

In order to investigate the in vitro drug release pattern, the dialysis bag technique was used as it is the most common technique utilized for determination of in vitro drug release from nano-carriers [52, 53]. A volume of optimized MZP-LNCs equivalent to 5 mg MZP was placed in a dialysis membrane (molecular weight cut-off 12,000–14,000 Da) previously soaked overnight in phosphate buffer (PB) of pH 6.8 [54–56]. Both ends of the membrane were knotted. To attain sink conditions, the bag was dipped in a well-sealed glass bottle containing 200 mL of PB of pH 6.8 to simulate the nasal fluid pH [57]. The bottle was then left in a shaking water bath at 100 rpm and 37 °C (Unimax, IKA, Germany). At predefined time intervals (0.5, 1, 2, 4, 6, 8, and 24 h), 3 mL of the release media was taken and an equal volume of fresh medium was promptly added to substitute the withdrawn samples. Then, the samples were filtered via a 0.45 μm membrane filter and assayed for MZP content spectrophotometrically at λ_max_ = 289 nm [10, 50]. A volume of MZP aqueous dispersion equivalent to 5 mg MZP was used as a control and passed through the same procedures to see if the dialysis cellulose membrane had any influence on the hinderance of drug release.

#### Stability study

The optimum formula was stored at 4 °C in an amber glass container to determine its stability. Zeta potential, particle size, polydispersity index and in vitro drug release were initially evaluated and then examined after storage for 3 months.

### In vivo biodistribution of the optimized MZP-LNCs

The in vivo biodistribution of **MZP-LNCs** was studied radiobiologically in mice utilizing radioiodinated MZP.

#### Preparation of radioiodinated MZP

##### Different conditions for ^131^I labeling of MZP

MZP was labeled with ^131^I by electrophilic substitution under various conditions (*n* = 3). Various factors should be studied before formulation of ^131^I-MZP including, N-chlorosuccinamide concentration, MZP concentration, reaction time and in vitro stability. We utilized varying quantities of MZP (30–600 µg) taken from a MZP ethanolic stock solution 99% (3:1, w/v), then variable quantities of N-chlorosuccinamide (20–220 μg) taken from N-chlorosuccinamide ethanolic stock solution 99% (2:1, w/v) were placed. For the labeling conditions, accurately 4 μL of ^131^I was employed. The components of the reaction were then vortexed. To stop the reaction, 4 μl of sodium metabisulphite solution (130 mg/mL) was added to make the excess iodine (I_2_) be reduced via turning it to iodide (I^−^) [58]. Additionally, in vitro stability was evaluated at room temperature.

The in vitro stability is another factor affecting the formed ^131^I-MZP, which establishes the ideal duration of time for injection to avoid formation of unwanted molecules generated from radiolysis [59]. The accuracy of biodistribution findings may be affected by these products as they might accumulate in undesirable organs [60]. Under the optimum conditions of preparation, where ^131^I-MZP was obtained and optimized, the in vitro stability test was conducted. The optimum reaction was left at room temperature for various time intervals, throughout that the samples radiochemical efficiency (% RCE) was assessed.

Origin 9.0 SR1 data analysis and graphics software version 90E (Copyright^©^, origin lab corporation, 1991–2013) was utilized to make electronic figures. Each factor was studied in triplicate experiments. The statistical analysis was done utilizing SPSS^®^ program, version 7.5.1 (SPSS Inc., Chicago, IL, USA). Data variances were investigated utilizing one-way ANOVA (significance level *p* ≤ 0.05). Data were stated as mean ± standard deviation.

##### Radiochemical efficiency assessment

The analytical methods of paper chromatography (PC) and thin layer chromatography (TLC) were employed to calculate radiochemical efficiency (RCE). For both PC and TLC, the utilized mobile phase was a mixture of ethanol: chloroform (1:9 v/v) that was freshly prepared. At the beginning line of the TLC strip, around 3 μL of the reaction mixture was put. The strip was removed after development, dried then sliced to 1 cm slices then investigated for radioactivity utilizing SR.7 gamma counter. TLC was used as well to determine radiochemical purity (RCP) [61].

The % radiochemical efficiency (RCE) was determined as the percentage ratio of ^131^I-MZP (labeled) activity in relation to the whole activity [62] as shown in the following equation:

1 $$\%\;\mathrm{RCE}=\frac{\mathrm{Activity}\;\mathrm{peak}\;\mathrm{of}\;{}^{131}\text{I}-\text{MZP}}{\mathrm{Total}\;\mathrm{activity}\;(\mathrm{free}\;{}^{131}\text{I}+\mathrm{labeled}\;{}^{131}\text{I}-\text{MZP})}\times100$$

#### Radio formulation of ^131^I-MZP-LNCs

^131^I-MZP-LNCs were formulated using the same method as before utilizing ^131^I -MZP instead of MZP then the final mixture was agitated for 5 min in a bath sonicator. The % RCE of ^131^I-MZP-LNCs was re-evaluated using chromatographical method to ensure the in vitro stability of the prepared ^131^I-MZP-LNCs.

#### In vivo biodistribution and pharmacokinetic studies

Animal study protocol was agreed by the Research Ethics Committee of Faculty of Pharmacy, Cairo University, Egypt (REC-FOPCU) with a PI (2728) reference number, and also approved by the animal ethics committee of the Labeled Compounds Department of the Egyptian Atomic Energy Authority (EAEA) Committee, Cairo, Egypt.

Biodistribution study for the optimized ^131^I-MZP-LNCs was utilized for evaluation of the in vivo behaviors of the optimized MZP-LNCs using 54 healthy Swiss albino male mice (weight 20–25g). At particular times, mice were kept at metabolic cages with water and food. At the time of the study, the mice were separated into 3 groups each containing 18 mice [63].

The administration of preparations was arranged as follows:

- Group I: Intravenous ^131^I-MZP-drug solution (IV-S) via mice tail vein
- Group II: Intranasal ^131^I-MZP drug solution (IN-S)
- Group III: Intranasal ^131^I-MZP-LNCs (IN-LNCs)

Each mouse received a volume with MZP dose equivalent to 2.5 ug/g of body weight. A Hamilton syringe attached to a polyethylene tube was used for IN administration into each mouse’s nostrils [64]. Mice were carefully set at a tilted posture during administration so that they could inhale the formulation. Three mice from every group were slaughtered at predefined time intervals (5, 15, 30, 60, 120, and 180 min post-delivery) [63]. Samples of blood were taken via heart punctures while the remaining organs involving brain were dissected, removed from sticking tissues and fluids, rinsed with normal saline, weighed, then their radioactivity caused by ^131^I-MZP absorption was determined using NaI gamma rays scintillation counter. As it was highly complicated to separate the whole of the mice’s blood, muscles and bones, their percentages were assumed to be 7, 40 and 10% of the animal’s overall weight, respectively [65–68]. Blood, bones and muscle samples were weighed to determine the entire weight of each of them. Percentage of the injected dose per gram (organ, fluids or tissue) (% ID/g) was utilized as an indication of radioactivity and it was determined at the predefined time intervals in a group including 3 mice utilizing the following equation [61, 69–71]:

2 $$\%\;\mathrm{ID}/\mathrm g=\frac{\mathrm{Activity}\;\mathrm{of}\;\mathrm{tissue}\;\mathrm{or}\;\mathrm{organ}}{\mathrm{Total}\;\mathrm{injected}\;\mathrm{activity}\times\mathrm{Weight}\;\mathrm{of}\;\mathrm{tissue}\;\mathrm{or}\;\mathrm{organ}}\times100$$

The pharmacokinetic parameters of MZP in every mouse were determined, including maximum ^131^I-MZP uptake (%ID/g) for brain and blood, T_max_ and C_max_. PKanalix 2023R program (Lixof SAS, Simulation Plus company, USA) was utilized for establishing the area under the concentration time curves from 0 to 180 min (AUC_0–180_ min %ID/g) as well as from 0 to ∞ (AUC_0-∞_ min %ID/g). Relative bioavailability for intranasal LNCs formed with ^131^I-MZP against ^131^I-MZP solution was calculated by applying the next equation [72]:

3 $$\mathrm{Relative}\;\mathrm{bioavailability}\;\%\;=\;\frac{(\mathrm A\mathrm U\mathrm C\;\mathrm L\mathrm N\mathrm C\mathrm s\;0-\infty)\mathrm{IN}}{(\mathrm A\mathrm U\mathrm C\;\mathrm s\mathrm o\mathrm l\mathrm u\mathrm t\mathrm i\mathrm o\mathrm n\;0-\infty)\text{IN}}\times100$$

Moreover, drug targeting efficiency (DTE) [52, 73, 74], drug targeting index (DTI) [75] as well as direct transport percentage (DTP) [76, 77] could wholly be used to depict the optimized MZP-LNC ability for brain targeting after intranasal administration [70]. Drug targeting efficiency % is the mean distribution proportion of the drug among brain and blood which was also calculated utilizing the equation below:

4 $$\mathrm{DTE}\;\%=\frac{\mathrm{AUC_{brain}\;IN}}{\mathrm{AUC_{blood}\;IN}}\times100$$

The following equation was used to calculate drug targeting index:

5 $$\mathrm{DTI}=\frac{\mathrm{AUC_{brain}}/\mathrm{AUC_{blood}\;IN}}{\mathrm{AUC_{brain}}/\mathrm{AUC_{blood}\;IV}}$$

where, AUC_brain_ indicates the area under the concentration versus time graph from 0 to 180 min for MZP in brain while AUC_blood_ indicates the area under the concentration versus time plot from 0 to 180 min for MZP in blood.

DTP% stands for the fraction of drug that transferred directly to the brain through the trigeminal and olfactory pathways and it is determined by the equation below:

6 $$\mathrm{DTP}\;\%=\frac{\mathrm{B_{IN}}-\mathrm{Bx}}{\mathrm{B_{IN}}}\times100$$

where, B_IN_ is the whole AUC_0–180 min_ in brain following IN delivery while Bx is a fraction of the systemic circulation’s AUC_0–180min_ in brain after IN delivery and it was determined by the equation below:

7 $$\mathrm{Bx}=\frac{\mathrm{B_{IV}}}{\mathrm{P_{IV}}}\times\mathrm{P_{IN}}$$

where, B_IV_ is the brain AUC_0–180min_ after IV administrative route, P_IV_ is the blood AUC_0–180min_ after intravenous administrative route and P_IN_ is the blood AUC_0–180min_ after IN administrative route.

## Results and discussion

### Formulation of MZP-LNCs utilizing D-optimal mixture design

Phase inversion technique was used to prepare LNCs. Oil in water emulsions is produced on low temperatures and water in oil emulsions is produced on elevated temperatures. When the system is exposed to repeated cooling and heating cycles while having a large surfactant content (> 10%w/w), lipid nanocapsules are formed on abrupt dilution by cold water. The lipid nanocapsules’ outer shell layer is formed because of the non-ionic surfactant’s shell crystallization that prevents globules from coalescing and results in stable lipid nanocapsule formation at room temperature [33, 78]. It is noteworthy that stability of mirtazapine will not be affected by heating as reported by several authors [79, 80].

The impact of the three independent variables, Labrafac % (X_1_), Solutol % (X_2_) and water% (X_3_); on the dependent variables, particle size (Y_1_), zeta potential (Y_2_), PDI (Y_3_) and solubilization capacity (Y_4_) were studied using D optimal mixture design. Table 2 shows the composition and responses of MZP-LNCs.

### Characterization of the prepared MZP-LNCs

#### Particle size and polydispersity index

As shown in Table 2, the particle diameter of MZP loaded lipid nanocapsules measured with zeta sizer varied from 18.74 to 131.5 nm. Thus, all the formulated LNCs were in the nanometer size which may be due to the presence of a large amount of lipoid phospholipids and surfactants [81]. Analysis of variance (ANOVA) was used to analyze the data and it showed that the special quartic model was the best model to correlate the particle size to the independent factors (Table 3**).** Analysis of variance shows that the model was statistically significant (*p* < 0.0001) and the lack of fit test was insignificant (*p* value 0.5628) where the probability value (α) to determine the statistical significance was considered at 0.05 level. The linear regression (*R*^2^) was equal to 0.9994 and the value of adequate precision was 123.26. Table 3 reveals the regression results correlating the particle size to the ternary mixture concentrations (X_1,_ X_2,_ X_3_). The term “adequate precision” determines the range of a predicted response in relation to its related error. It determines the signal to noise ratio where ratios larger than four are considered appropriate to navigate any design space [82]. So, the adequate precision value of 123.26 reveals model’s great suitability. Additionally, the model’s nonsignificant lack of fit indicates that the data is fitted on the studied model.

**Table 3:** Regression analysis of the measured responses of MZP-LNCs depending on the optimum model

Response	Model	*R*^2^	Adjusted *R*^2^	Predicted *R*^2^	Adequate precision	PRESS	Significant terms	Regression equation of the responses
Y_1_: particle size	Special quartic	0.9994	0.9987	NA*	123.2628	NA*	X_1_, X_2_, X_3_, X_1_X_2_	Particle size = 73.82 X_1_ + 19.73 X_2_ + 23.84 X_3_ -52.46 X_1_X_2_
Y_3_: PDI	Quadratic	0.8687	0.8090	0.6688	9.7572	3.38	X_2_, X_3_, X_1_X_2,_ X_1_X_3_	1/sqrt (PDI) = 2.15 X_2_ + 3.45 X_3_ + 9.01 X_1_X_2_ + 7.27 X_1_X_3_
Y_4_: Solubilization capacity	Reduced Cubic	0.9938	0.9901	0.9847	51.6952	0.5363	X_1_, X_2_, X_3_, X_1_X_2,_ X_1_X_3,_ X_2_X_3,_ X_2_X_3_(X_2_-X_3_)	Solubilization capacity = 4.8 X_1_ + 7.08 X_2_ + 2.19 X_3_ -1.71 X_1_X_2_ -1.43 X_1_X_3_ -5.62 X_2_X_3_ -3.56 X_2_X_3_(X_2_-X_3_)

The model of Y_2_ (zeta potential) was statistically insignificant (*p* > 0.05) and excluded from optimization., NA* means statistically not defined

**Fig. 1:**
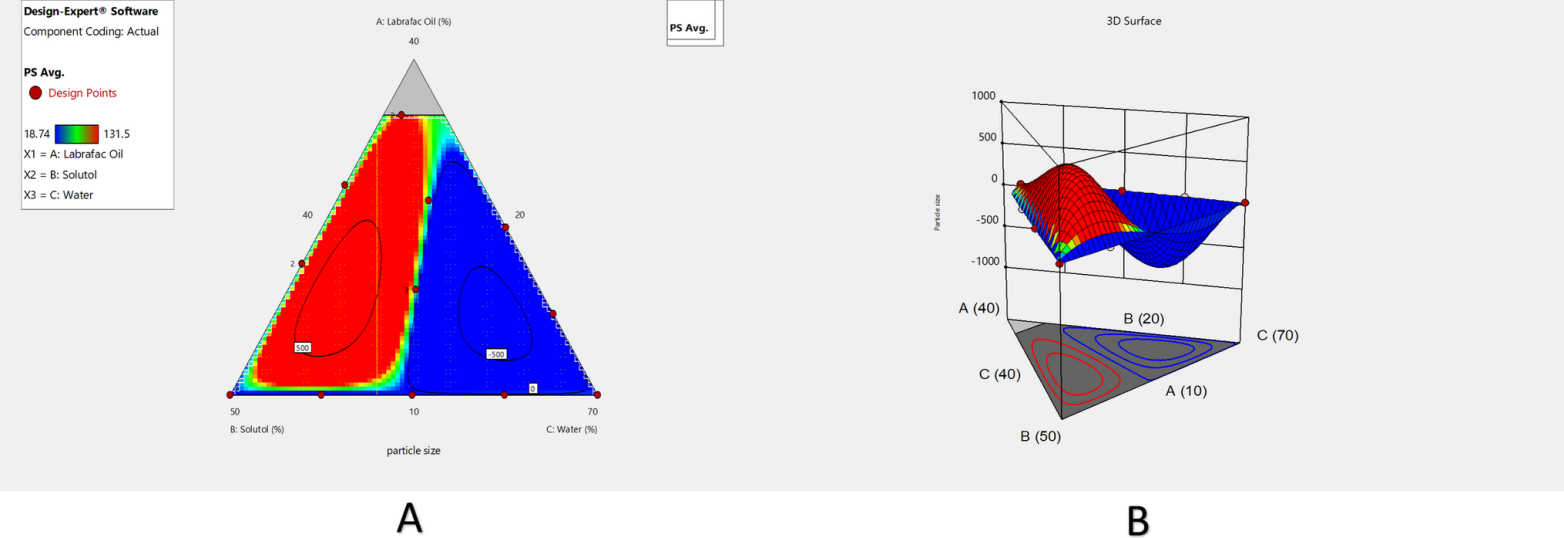
Contour plot (**A**) and 3D-Response surface plot (**B**) viewing the effect of Labrafac, Solutol and water on the particle size of MZP-LNCs

Statistical analysis revealed that the X_1_, X_2_, X_3_ as well as interaction term X_1_X_2_ had significantly affected particle size (*p* < 0.05) (Table 3). Figure 1A (contour plot) and B (response surface plot) represent the effects of surfactant, oil as well as water on the particle size of MZP-LNCs. Increasing oil percentage would increase the particle size as the oil makes up the core of lipid nanocapsules. Thus, when the oil percentage increases, the core size increases and the particle size will consequently increase [48]. Regarding the effect of surfactant concentration, increasing surfactant percentage would decrease the particle size at low surfactant concentration levels. Surfactants have inherent solubility in water as well as in oil and therefore, they are arranged at the oil water interface causing a decrease in the surface tension and subsequent reduction in particle size [48, 81, 83]. However, at high surfactant concentration levels, increasing its percentage would increase the particle size because the higher surfactant percentage may cause particle aggregation [84]. The significant interaction effect of X_1_ (Labrafac) and X_2_ (Solutol) on the particle size appears at intermediate concentration levels of water (X_3_). Thus, at low level of Labrafac concentrations and high level of Solutol concentrations, large particle size will be obtained. Afterwards, increasing concentration of Labrafac and decreasing concentration of Solutol will result in gradual decrease in particle size. Upon reaching the higher level of Labrafac concentration and lower level of Solutol concentration, particle size will gradually increase again.

**Fig. 2:**
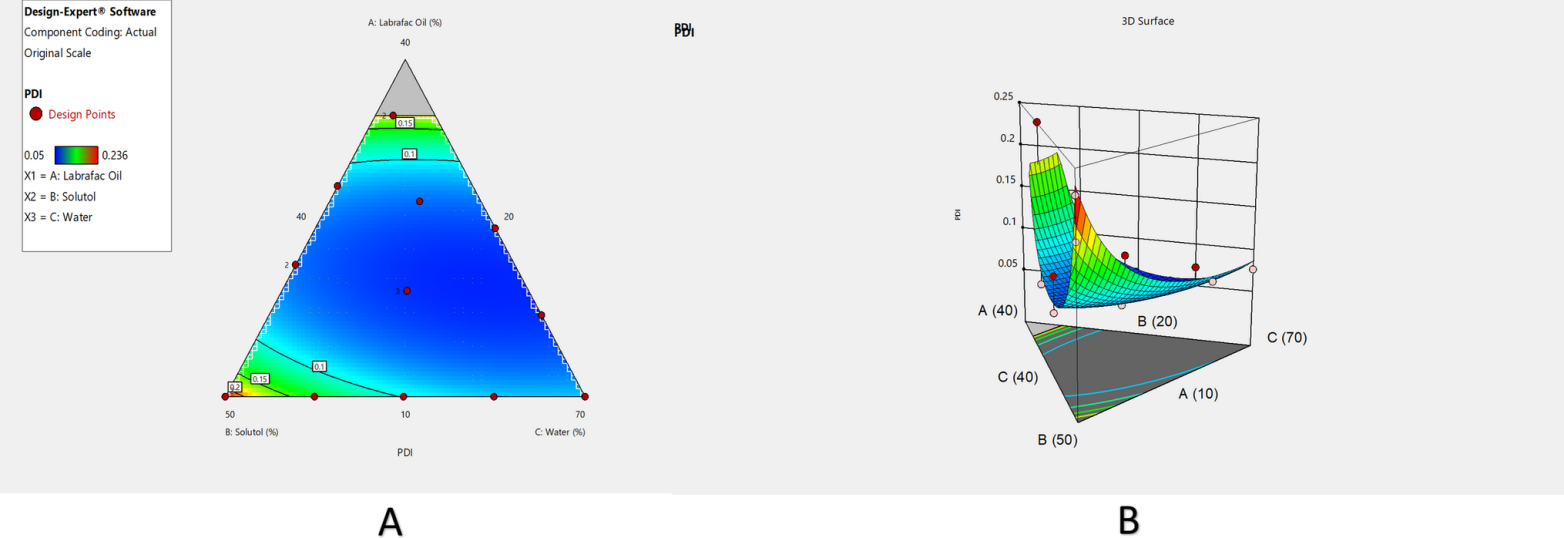
Contour plot (**A**) and 3D-Response surface plot (**B**) viewing the effect of Labrafac, Solutol and water on the polydispersity index of MZP-LNCs

As shown in Table 2, MZP-LNCs had repeatable narrow particle sizes as well as monomodal homogenous distribution. The majority of PDI values are less than 0.2 which confirmed that the particle size obtained by the adopted method of preparation was homogenous and uniform [85, 86]. ANOVA for quadratic model of polydispersity index shows the statistical significance of the model (*p* value 0.0002). Regression results of the PDI are revealed in Table 3. Statistical analysis showed that X_2_, X_3_, the interaction terms X_1_X_2_ and X_1_X_3_ had significant effects on PDI (*p* < 0.05) (Table 3). The effects of water, surfactant as well as oil on polydispersity index of MZP-LNCs are presented graphically in Fig. 2A (contour plot) and B (response surface plot). Table 3 describes the polydispersity index prediction equation. The optimal zone exhibiting good system homogeneity is shown in the high as well as the intermediate levels of surfactant percentage. Being a surfactant, Solutol molecules are arranged at the water–oil interface decreasing interfacial tension which results in small as well as homogenous particle sizes [81, 87].

#### Zeta potential

The prepared lipid nanocapsules carried a negative charge where the zeta potential values ranged between − 3.02 and − 7.6 mV. This may be due to the effect of the negative charge of the phospholipid [36, 88] as well as the Solutol which could conduct the negative charge to the particles due to the existence of PEG dipoles [89]. ANOVA for special quartic model of the zeta potential indicated the statistical insignificance of the model (*p* value 0.0624). However, ANOVA for zeta potential showed that the combined effects of the independent factors were statistically significant (*p* < 0.05). Thus, the interaction between the independent factors was due to the surface charge of the particles rather than the single effect of every independent factor.

#### Solubilization capacity of MZP-LNCs

**Fig. 3:**
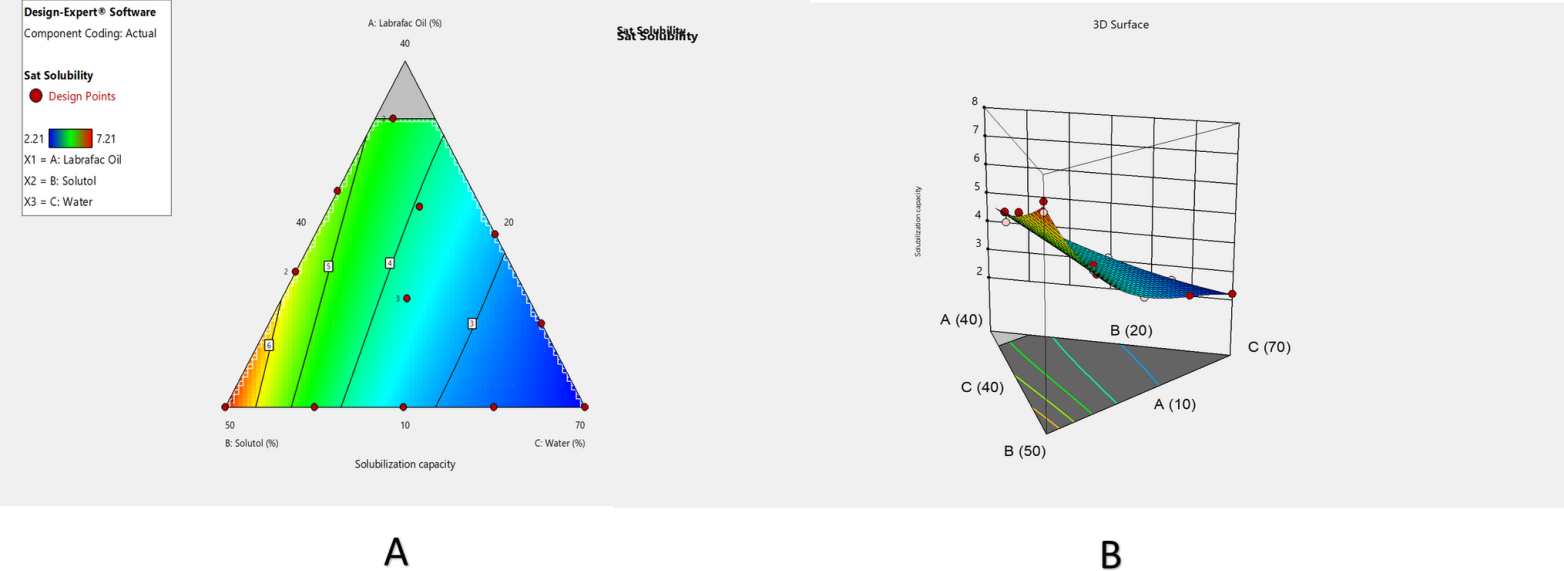
Contour plot (**A**) and 3D-response surface plot (**B**) viewing the effect of Labrafac, Solutol and water on the solubilization capacity of MZP-LNCs

A solubilization capacity test was established for identifying the variables influencing MZP solubility to increase the drug payload in lipid nanocapsule formulations as well as to enhance its suitability for intranasal delivery. As shown in Table 2, the resulted MZP solubilization values varied between 2.21 and 7.21 mg/g in lipid nanocapsule systems. Reduced cubic model was the best model to correlate MZP solubilization to the ternary mixture concentrations. Results showed the statistical significance of the model (*p* < 0.0001). Table 3 revealed the regression results of solubilization capacity (Y_4_) as well as the equation that correlated MZP solubilization to the independent variables. Contour plot and 3D response surface plot (Fig. 3A and B) show that there was a direct relationship between MZP solubilization and the Labrafac as well as Solutol percentages. Large concentrations of Labrafac as well as Solutol improved MZP solubilization efficiency of MZP which is quite logical as MZP has poor water solubility.

### Formulation optimization of MZP-LNCs

**Fig. 4:**
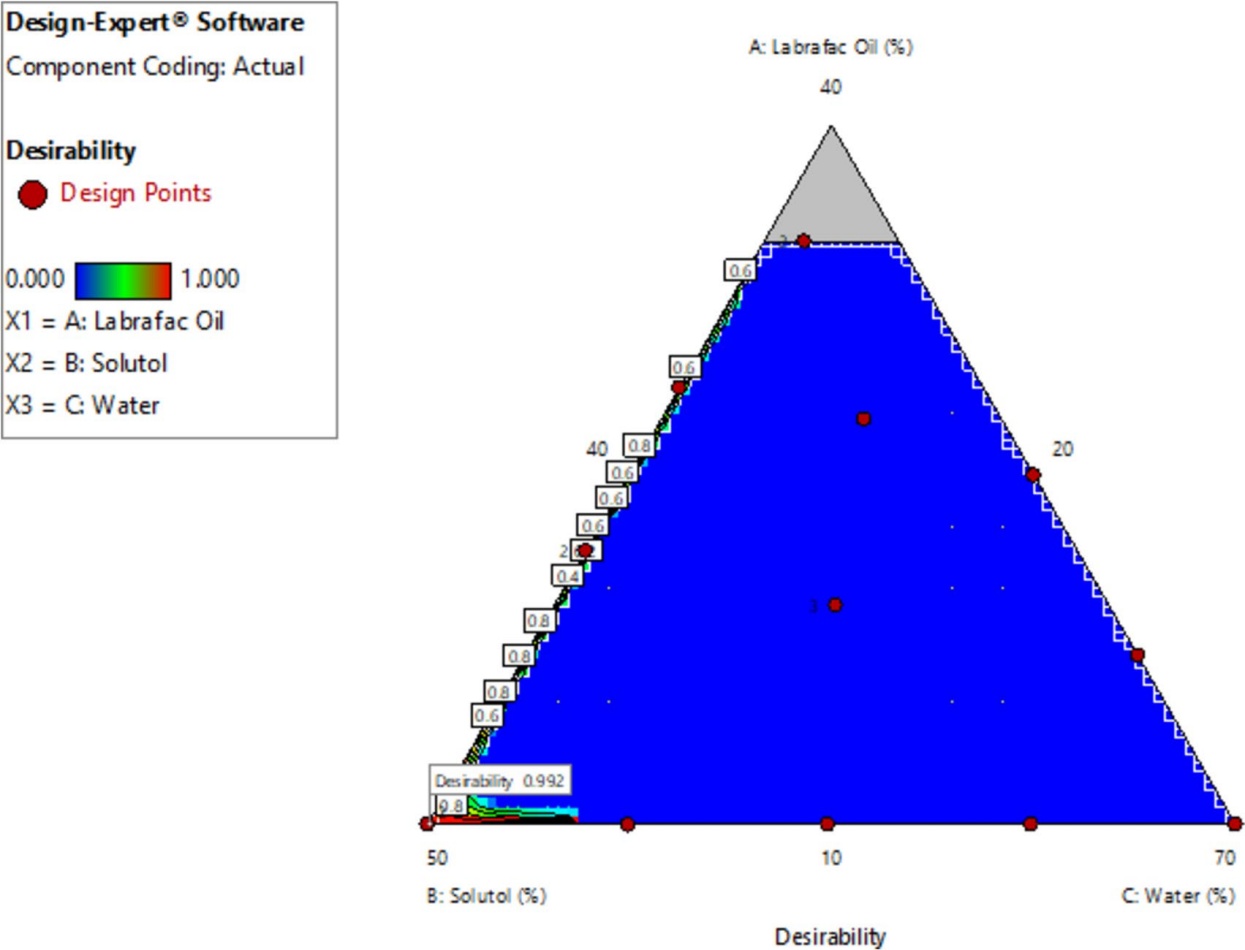
Contour plot positioning the location of the optimum MZP-LNCs in the design space showing its desirability

The goal of the optimization process is to determine the ideal level of each factor needed to prepare a high-quality pharmaceutical product. Design Expert^®^ program was utilized in this study to provide a numerical optimization method, utilizing the desirability function for overcoming the multiple as well as the opposing responses [90]. Design Expert^®^ software proposed many MZP-LNCs formulae in accordance with particle size, zeta potential, polydispersity index and solubilization capacity results. Zeta potential model (Y_2_) was statistically insignificant (*p* > 0.05), so, it wasn’t included in the optimization process. The optimization criteria to select the optimum formula were to minimize PS, PDI and maximize solubilization capacity. For each formulation, design expert proposed desirability varying from 0 to 1 in accordance with its responses. The optimized MZP-LNCs is composed of 10% Labrafac, 50% Solutol and 40% water and it had a maximum desirability of 0.992 in the design space as expected by mathematical modelling (Fig. 4).

### Evaluation of the optimized MZP-LNCs

The optimized MZP-LNC which was predicted by D optimal mixture design was prepared and evaluated as the previously formulated LNCs in order to assess the efficacy of the optimization process. The observed values of the proposed formula were compared to the predicted values for all responses. Also, residual value that corresponds to the differences between the observed and predicted value for every response was calculated as illustrated in Table 4. All responses had small residual values being not more than 0.861, indicating that the process of the optimization was reasonable. Hence, the proposed MZP-LNCs was selected as the optimized formula and employed for more investigation.

**Table 4:** Optimum levels of independent factors of the proposed MZP-LNCs formula with the predicted, observed and residual values of all responses

Factor		Optimum level (%)	
X_1_: Labrafac		10	
X_2_: Solutol		50	
X_3_: Water		40	

Response	Predicted value	Observed value	Residual value^a^
Y_1_: Particle size (nm)	19.729	20.59	− 0.861
Y_2_: Zeta potential (mV)	− 5.919	− 5.71	− 0.209
Y_3_: Polydispersity index	0.216	0.223	− 0.007
Y_4_: Solubilization capacity (mg/g)	7.077	7.21	− 0.133

^a^Residual value = predicted value-observed value

#### Transmission electron microscopy (TEM)

**Fig. 5:**
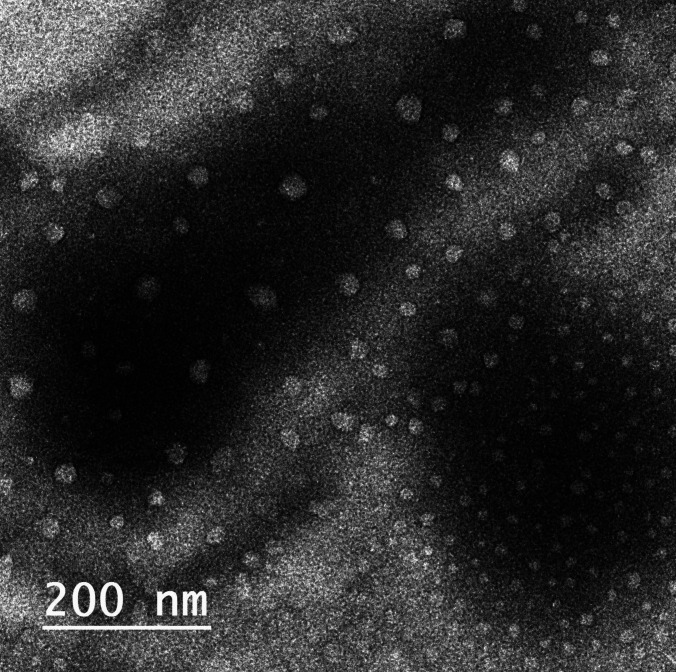
Transmission electron micrograph of optimum MZP-LNCs

TEM micrograph (Fig. 5) showed spherical shape of the optimized MZP-LNCs. It confirmed the size uniformity of nanocapsules without any aggregation. The particle size obtained from transmission electron microscope and that determined with zeta sizer were almost the same.

#### In vitro release study

**Fig. 6:**
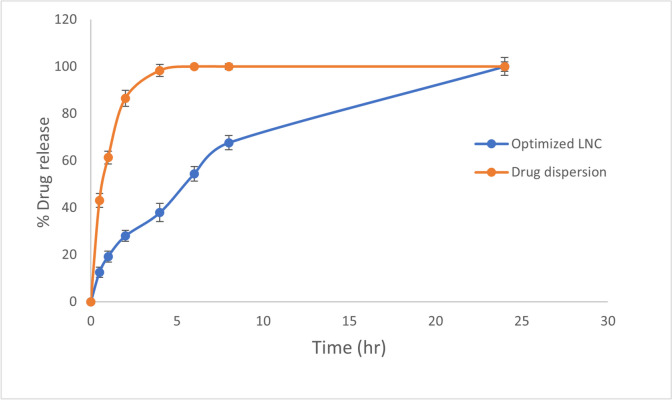
In vitro MZP release from optimized MZP-LNCs in comparison with the drug dispersion in phosphate buffer of pH 6.8 (Results are presented as mean ± SD, *n* = 3)

The in vitro release profiles of MZP from the optimum MZP-LNCs compared to drug aqueous dispersion are presented in Fig. 6. MZP aqueous dispersion release profile showed fast release of the drug, of around 100% ± 1% drug release after 6 h. This supposed that the drug freely diffused across the dialysis membrane, revealing that the membrane utilized didn’t prevent the drug from reaching the release medium. Conversely, the optimized MZP-LNCs release profile was characterized by a slower release rate (54.38% ± 3.1 after 6 h) than MZP aqueous dispersion which was quite reasonable, since the oil content in the LNCs may impede the rate of MZP diffusion into the release medium. Thus, the slow diffusion behavior of the drug lipid nanocapsules might have an important effect on decreasing the frequency of MZP daily administration in comparison with MZP dispersion.

#### Stability study

The optimized MZP-LNC showed good physical stability when stored for 3 months at 4 °C. Thus, the original particle size was 20.59 nm while it was 21.16. nm after storage for 3 months. The original polydispersity index was 0.223 while it was 0.227 following storage for 3 months. Moreover, no change was detected in the negative zeta potential of the optimum MZP-LNCs upon storage. The in vitro release study results for the optimum formula, initial as well as following the storage period revealed slow-release behavior in comparison with MZP aqueous dispersion that achieved 100 ± 1% MZP release after 6 h. The similarity between the original MZP release and that following 3-month storage was determined using the similarity factor equation [91]. The determined similarity factor was 48 and this confirmed that the optimum MZP-LNCs formula was very stable and gave a robust release profile which remained unchanged over the storage period.

### Preparation of radioiodinated MZP

**Fig. 7:**
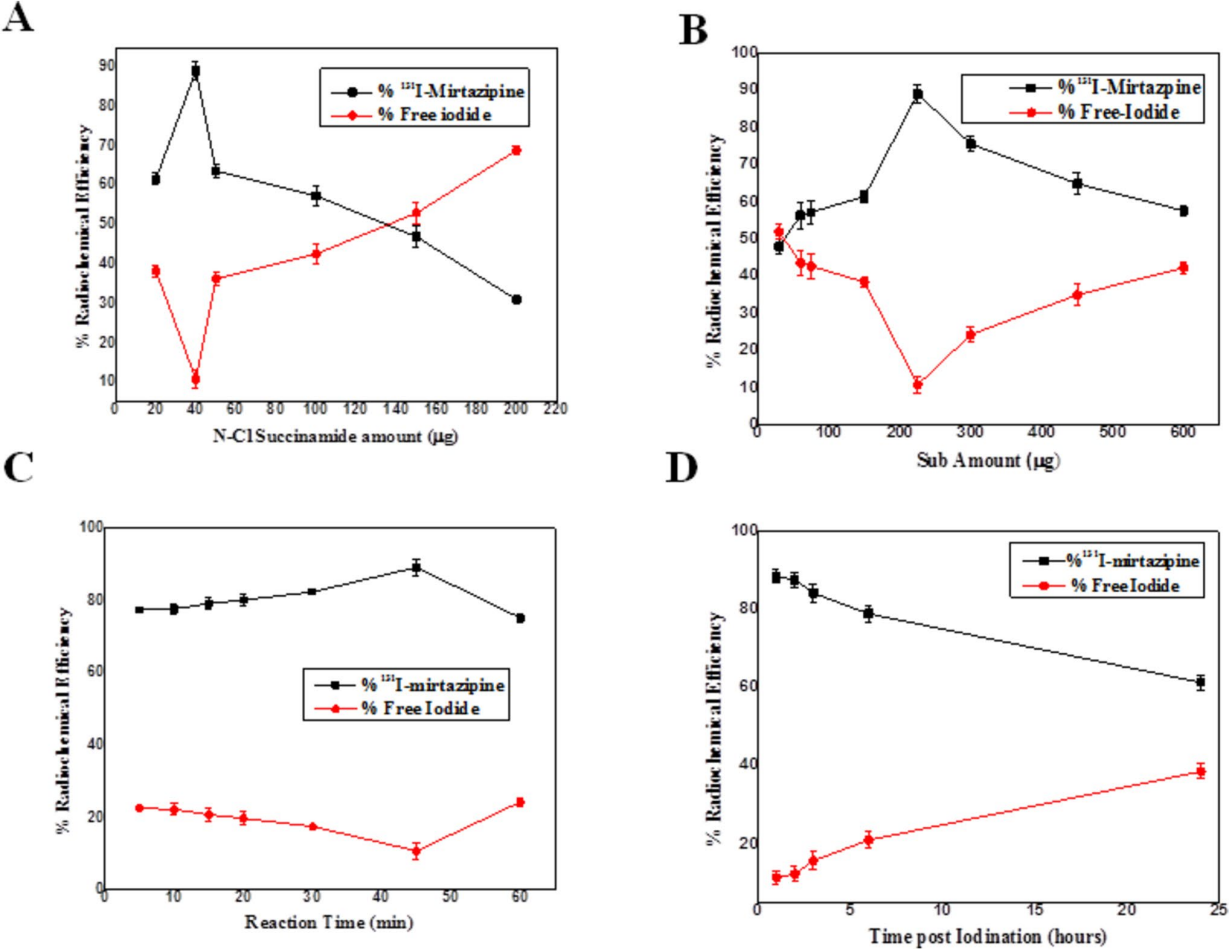
Variation of the radiochemical efficiency % of ^131^I-MZP as a function of **A** N-Cl succinamide (NCS) concentration (µg); working conditions: 4 µL Na^131^I for 45 min, 225 µg of MZP, x µg of NCS, **B** substrate (MZP) concentration (µg); working conditions: x µg of MZP, 40 µg of NCS, 4 µL of Na^131^I for 45 min, **C** reaction time (min); working conditions: 4 µL of Na^131^I for x minutes, 225 µg of MZP, 40 µg of NCS and **D** time post iodination (hour) (in vitro stability); working conditions: 225 µg of MZP, 40 µg of NCS, 4 µL of Na^131^I for 45 min

Direct electrophilic substitution method with the presence of an oxidizer (NCS) was used for radio-iodination of MZP [92]. After the study of the abovementioned parameters that influenced the reaction, the amount of the oxidizing agent was found to be the most important factor in determining the RCE as it permits an electrophilic reaction to occur through production of iodonium ion from iodide ion. This important step was accomplished accurately at 40 ug of NCS (Fig. 7A). Deviations from 40 ug NCS could cause a strong decrease in RCE. This decrease might happen because of unfavorable waste products’ production with large NCS quantities or because of inadequate radioactive iodine’s oxidation with small NCS quantities [93, 94]. The highest RCE% was achieved at 225 μg of MZP (Fig. 7B). RCE decreases with smaller MZP quantity which might happen because there aren’t enough accessible substrate that is needed for catching all of iodonium in the solution. Reaction time, which could be completed in 45 min (Fig. 7C), was another important factor in achieving the highest %RCE. Finally, the formed ^131^I-MZP was stable for 3 h after iodination (Fig. 7D). So, the maximum RCE of 89.2% ± 2.4 was attained through adding 4 uL Na^131^I into a mixture of 225 ug MZP as well as 40 ug NCS for 45 min in 1 mL of total reaction volume.

#### Radio formulation of ^131^I-MZP-LNCs

The ^131^I-MZP was prepared with the highest %RCE before being loaded onto the optimized LNCs. To ensure the formula’s in vitro stability, RCE of ^131^I-MZP-LNCs was reevaluated using chromatographic method. Results demonstrated that the formula was stable having large RCE (> 85%).

#### In vivo biodistribution and pharmacokinetic studies

**Fig. 8:**
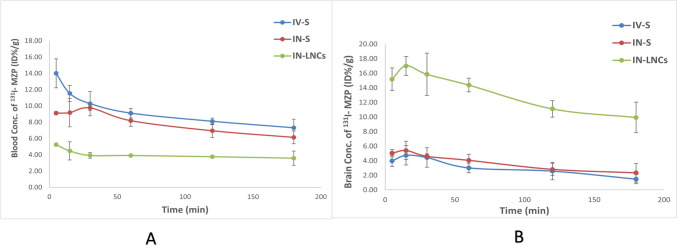
Blood (**A**) and Brain (**B**) MZP concentrations (%ID/g) at various time intervals following administration of intravenous ^131^I-MZP-drug solution (IV-S), intranasal ^131^I-MZP drug solution (IN-S) and intranasal ^131^I-MZP-LNCs (IN-LNCs) (mean ± SD, *n* = 3)

Table 5 shows the uptake of radioiodinated MZP in brain and blood over 180 min in the three experimental animal groups. Figure 8A and B reveals ^131^I-MZP concentration in blood and brain respectively, following administration of various radioiodinated MZP treatments. Group III (IN-LNCs) showed significantly greater ^131^I-MZP brain concentration (17.02 ± 1.61%ID/g at 15 min after administration) compared to the other two groups Group I (IV-S) and Group II (IN-S) (4.75 ± 1.32%ID/g and 5.41 ± 1.27%ID/g at 15 min after administration, respectively) (*p* < 0.05). On the other hand, Group I showed greater blood ^131^I-MZP concentration compared to the other two groups and that was owing to the direct transport of the drug to systemic circulation after intravenous administration. The plasma concentration of ^131^I-MZP was lower in group III (IN-LNCs) which will reflect a decrease in side effects when compared to ^131^I-MZP solutions (IV-S and IN-S). The discrepancies between the three groups’ mean blood and brain uptakes were then compared (*p* ≤ 0.05), and it was found that IN-LNCs was significantly different from IV-S and IN-S but that there was non-significant difference between IV-S and IN-S.

**Table 5:** MZP concentrations in brain, blood as well as brain/blood ratios of MZP different formulations (intravenous ^131^I-MZP-drug solution (IV-S), intranasal ^131^I-MZP drug solution (IN-S) and intranasal ^131^I-MZP-LNCs (IN-LNCs)) in mice (mean ± SD, *n* = 3)

Administrative route of radioiodinated formulations	Organ/Tissue	% ID/g					
		5 min	15 min	30 min	60 min	120 min	180 min
IV-S	Blood	14.0 ± 1.78	11.5 ± 0.98	10.28 ± 1.49	9.11 ± 0.58	8.11 ± 0.39	7.32 ± 1.06
	Brain	3.98 ± 0.76	4.75 ± 1.32	4.45 ± 1.34	3.02 ± 0.67	2.58 ± 1.2	1.48 ± 0.63
	Brain/blood	0.29 ± 0.03	0.39 ± 0.02	0.42 ± 0.02	0.32 ± 0.05	0.3 ± 0.07	0.19 ± 0.01
IN-S	Blood	9.12 ± 0.21	9.18 ± 1.74	9.76 ± 0.35	8.19 ± 0.73	6.96 ± 0.83	6.14 ± 0.79
	Brain	5.04 ± 0.51	5.41 ± 1.27	4.62 ± 0.33	4.06 ± 0.84	2.82 ± 0.83	2.35 ± 1.27
	Brain/blood	0.54 ± 0.07	0.6 ± 0.09	0.46 ± 0.04	0.5 ± 0.06	0.41 ± 0.05	0.37 ± 0.04
IN-LNCs	Blood	5.25 ± 0.11	4.48 ± 1.11	3.92 ± 0.35	3.91 ± 0.1	3.77 ± 0.15	3.58 ± 0.87
	Brain	15.18 ± 1.72	17.02 ± 1.61	15.86 ± 0.57	14.38 ± 1.21	11.12 ± 2.01	9.95 ± 0.2
	Brain/blood	2.3 ± 0.1	3.9 ± 0.5	4.2 ± 0.6	3.9 ± 0.4	3.1 ± 0.3	2.8 ± 0.1

**Fig. 9:**
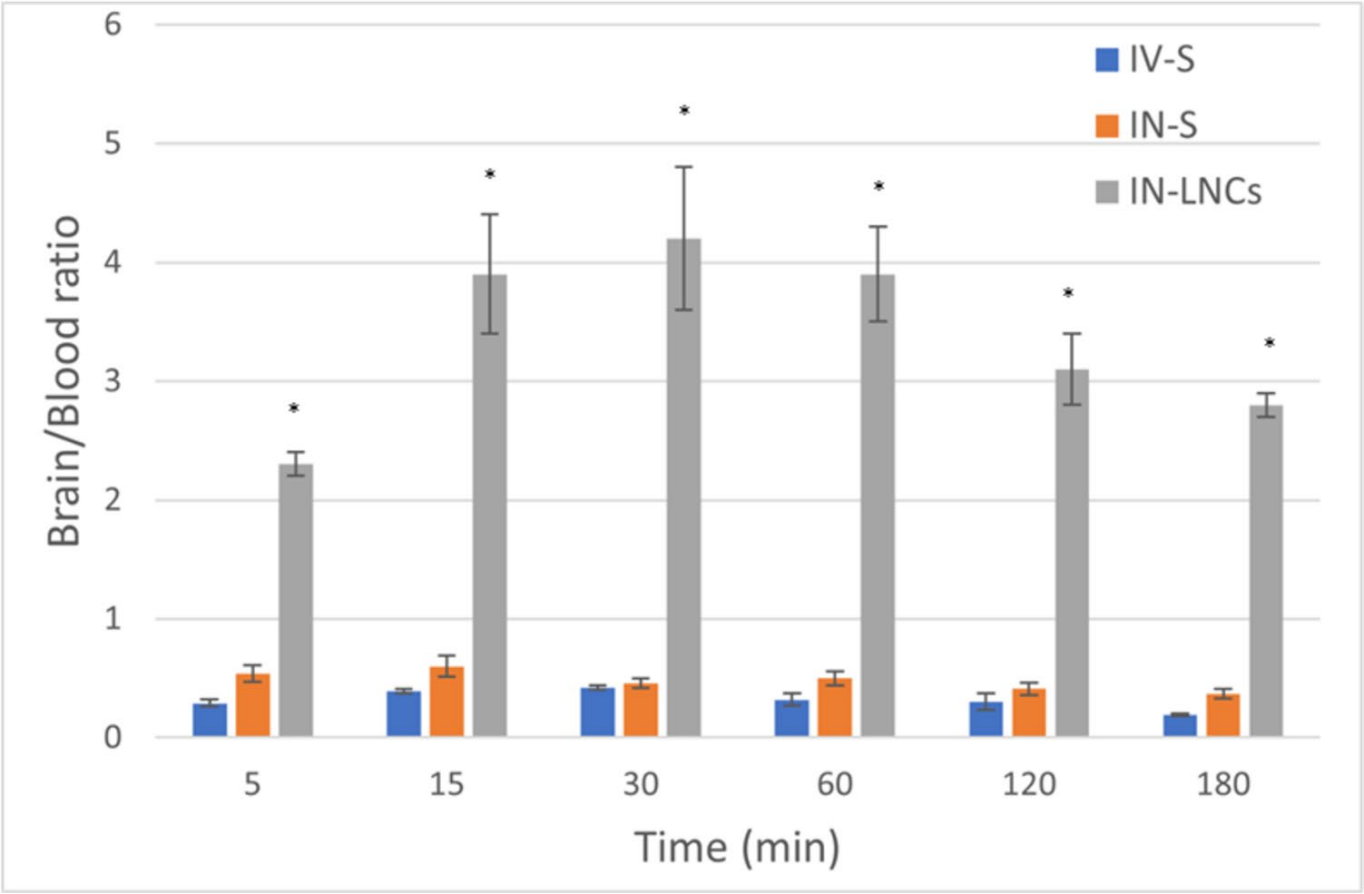
Brain/blood concentration ratios of mirtazapine following administration of intravenous ^131^I-MZP-drug solution (IV-S), intranasal ^131^I-MZP drug solution (IN-S) and intranasal ^131^I-MZP-LNCs (IN-LNCs) (mean ± SD, *n* = 3) (*) means significant difference (*p* < 0.05)

The brain/blood ratios of different radiolabeled MZP preparations (Table 5, Fig. 9) were calculated by dividing the brain ^131^I-MZP concentration values by the blood values for every mouse at equal time intervals. Group III showed significant higher brain/blood ratios (*p* < 0.05) in comparison with the other two groups, indicating the enhanced LNC formulation’s capability to target the brain.

The three administered radioiodinated formulae’s pharmacokinetic parameters were calculated (AUC_0–180_, AUC_0–∞,_ T_max_ and C_max_) for brain and blood and the results are listed in Table 6. IN-LNCs showed significant greater AUC_0–∞_ and C_max_ in brain confirming the capability of LNCs to deliver MZP and target it directly to the brain.

**Table 6:** Blood and brain pharmacokinetic parameters of MZP after administration of intravenous ^131^I-MZP-drug solution (IV-S), intranasal ^131^I-MZP drug solution (IN-S) and intranasal ^131^I-MZP-LNCs (IN-LNCs) (mean ± SD, *n* = 3)

Formulation/route of administration	Organ/Tissue	C_max_ (%ID/g)	T_max_ (min)	AUC_0-∞_ (min %ID/g)	AUC_0–180_ (min %ID/g)	Relative bioavailability %
IV-S	Blood	13.37 ± 2.15	10	5785 ± 560.17	1554.2 ± 49.31	
	Brain	5.06 ± 1.04	10	743.84 ± 91.74	480.96 ± 61.52	
IN-S	Blood	10.7 ± 1.33	10	4038.1 ± 742.52	1350.4 ± 99.7	
	Brain	5.83 ± 0.83	10	1007.22 ± 63.93	619.24 ± 95.16	
IN-LNCs	Blood	5.32 ± 0.19	10	2162.7 ± 204.61	679.86 ± 39.76	53.56
	Brain	17.52 ± 1.11	10	5129.56 ± 456.58	2258.47 ± 187.8	509.28

The nasal cavity is composed mainly of two regions which are the olfactory and the respiratory regions. The respiratory region is greatly vascularized, whereas the olfactory region is stimulated by the olfactory nerve [16]. There are two pathways through which the drug can enter the brain after intranasal administration as clarified by earlier studies [56]. The first one is the absorption of the drug from the respiratory region of the nasal cavity across the nasal mucosa to the systemic circulation, then crossing the blood brain barrier to enter the brain. A previous study was done and it was successful in improving nimodipine delivery to the brain via penetration of the BBB from systemic circulation following IN administration of nimodipine loaded LNCs [48]. On the other hand, in the second pathway (direct nose to brain pathway), the drug is transferred from nasal cavity’s olfactory region into the olfactory bulb and then directly to the brain [95]. In this study, we successfully improved mirtazapine transport to the brain via the second pathway, bypassing the BBB, following IN delivery of MZP-LNCs. Direct nose to brain pathway in order to bypass the blood brain barrier and transport drugs directly into the brain is of significant interest [96], even though the olfactory region just comprises around 5% of the entire volume of the nasal cavity [97]. Moreover, there are 2 various ways through which drugs can be transferred to the olfactory bulb from the olfactory area and subsequently to several sections in brain. Those ways are the intracellular and extracellular pathways. Concerning the intracellular pathway, the olfactory neurons take in the drug which is then emitted via exocytosis from the projection region of the neurons. On the other hand, at extracellular mechanism, the drug primarily passes the nasal epithelium into the lamina propria region which contains neurons in the olfactory region [95].

According to the pharmacokinetic data obtained, optimized MZP loaded LNCs had a greater capability to deliver MZP directly to the brain after IN administration. These outcomes were also proved by mathematical calculation of specified parameters which were DTP, DTI, DTE as well as the relative bioavailability (RB) [98]. The determined RB of IN-LNCs was 53.56% and 509.28% for blood and brain, respectively as displayed in Table 6. Moreover, the values of DTE%, DTI and DTP% for IN-LNCs were 332.2%, 10.73 and 90.68% respectively. On the other hand, these values for IN-S were 45.86%, 1.48 and 32.52% respectively as shown in Table 7. DTE is the partitioning time average of the ^131^I-MZP among the brain and blood [28]. The higher value of DTE calculated for IN-LNCs indicates that IN-LNCs displayed quicker as well as higher brain delivery in comparison with IN-S [77]. DTP represents the percentage of ^131^I-MZP transferred directly to the brain through the olfactory or trigeminal nerve [70, 77]. The greater DTE and DTP values for IN-LNCs compared with those for IN-S could justify the greater penetration of LNCs into the nasal mucosa to target the brain effectively [20, 99, 100]. DTI implies the extent of drug brain targeting after IN delivery. Hence, the greater DTI value for IN-LNCs compared with that for IN-S confirmed that the former provided more efficient MZP brain delivery and targeting than the later [77, 101]. The results of %RB also proved that MZP was directly transported to the brain from the nose through IN-LNCs.

**Table 7:** Drug targeting efficiency (DTE), drug targeting index (DTI), and direct transport percentage (DTP) of intranasal ^131^I-MZP drug solution (IN-S) and intranasal ^131^I-MZP-LNCs (IN-LNCs) in comparison with intravenous ^131^I-MZP-drug solution (IV-S)

Formulation/route of administration	DTE (%)	DTI	DTP (%)
IN-S	45.86	1.48	32.52
IN-LNCs	332.2	10.73	90.68

All these results can be attributed to the tiny particle size as well as flexibility of the prepared LNCs. These features support the delivery of the drug to the brain by intracellular as well as extracellular pathways, as declared before. The olfactory neurons were able to ingest MZP-LNCs due to their tiny size (intracellular route), while their flexibility helped them to squeeze and cross via the narrow extracellular pathway directly to brain [102]. Several studies have been reported for targeting drugs to the brain upon IN administration when loaded into nano-carriers [56, 103–108].

## Conclusion

In this work, mirtazapine-loaded lipid nanocapsules (MZP-LNCs) were effectively fabricated utilizing solvent-free phase inversion method for brain transport directly from the nasal cavity. The D-optimal mixture design was applied to study the impact of different formulation variables on the characterization of the formulated LNCs. The optimized formula loaded with MZP showed excellent properties with small particle diameter, homogenous size distribution, negative zeta potential and spherical shape as confirmed by transmission electron microscopy. In vivo biodistribution of the drug in mice was assessed by a radiobiological technique using radioiodinated mirtazapine (^131^I-MZP). Results of these studies confirmed that the optimum formulation achieved successful brain targeting with lower drug levels in blood after IN administration compared to IV and IN drug solutions as indicated by the high DTP, DTI and DTE values of such formula. The differences between the mean blood and brain uptakes of the optimum formula after its IN administration and the drug solution after its IV or IN administration was found to be statistically significant at *p* ≤ 0.05, while such difference was found to be non-significant upon comparing the IV with the IN administration of the drug solution.

As a final conclusion, the obtainable findings suggest that IN administrative route of MZP loaded LNCs is a favorable method to enhance brain drug targeting.

## Data Availability

The datasets generated during and/or analyzed during the current study are available from the corresponding author on reasonable request.
